# Bionanocomposite Blown Films: Insights on the Rheological and Mechanical Behavior

**DOI:** 10.3390/polym13071167

**Published:** 2021-04-05

**Authors:** Maria Chiara Mistretta, Luigi Botta, Rossella Arrigo, Francesco Leto, Giulio Malucelli, Francesco Paolo La Mantia

**Affiliations:** 1 Dipartimento di Ingegneria, Università di Palermo, Viale Delle Scienze, 90128 Palermo, Italy; mariachiara.mistretta@unipa.it (M.C.M.); luigi.botta@unipa.it (L.B.); francesco.leto@hotmail.it (F.L.); 2 Consorzio Interuniversitario per la Scienza e Tecnologia dei Materiali, INSTM, Via Giusti 9, 50121 Firenze, Italy; rossella.arrigo@polito.it (R.A.); giulio.malucelli@polito.it (G.M.); 3 Dipartimento di Scienza Applicata e Tecnologia, Politecnico di Torino, Viale Teresa Michel 5, 15121 Alessandria, Italy

**Keywords:** biopolymers, bionanocomposites, nanoclays, rheological behavior, mechanical properties, film blowing process

## Abstract

In this work, bionanocomposites based on two different types of biopolymers belonging to the MaterBi^®^ family and containing two kinds of modified nanoclays were compounded in a twin-screw extruder and then subjected to a film blowing process, aiming at obtaining sustainable films potentially suitable for packaging applications. The preliminary characterization of the extruded bionanocomposites allowed establishing some correlations between the obtained morphology and the material rheological and mechanical behavior. More specifically, the morphological analysis showed that, regardless of the type of biopolymeric matrix, a homogeneous nanofiller dispersion was achieved; furthermore, the established biopolymer/nanofiller interactions caused a restrain of the dynamics of the biopolymer chains, thus inducing a significant modification of the material rheological response, which involves the appearance of an apparent yield stress and the amplification of the elastic feature of the viscoelastic behavior. Besides, the rheological characterization under non-isothermal elongational flow revealed a marginal effect of the embedded nanofillers on the biopolymers behavior, thus indicating their suitability for film blowing processing. Additionally, the processing behavior of the bionanocomposites was evaluated and compared to that of similar systems based on a low-density polyethylene matrix: this way, it was possible to identify the most suitable materials for film blowing operations. Finally, the assessment of the mechanical properties of the produced blown films documented the potential exploitation of the selected materials for packaging applications, also at an industrial level.

## 1. Introduction

Polymers and polymers-based systems are currently widely exploited for packaging applications, because of their low cost, ease of processability, and tunable properties, which can be properly tailored depending on the product requirements [1,2,3]. In fact, the morphology and the mechanical, thermal, and optical properties of polymeric materials can be profitably modified through chemical functionalization methods [4], melt blending [5,6] or by introducing several kinds of micro- to nano-sized fillers [7,8,9], resulting in a huge variety of finished packaging materials with superior mechanical strength, transparency, and barrier properties towards different gases [10,11].

Polyethylene, polypropylene, polyethylene terephthalate, and polystyrene are the most common polymers employed in the packaging industry, accounting for more than 90% of the total volume of plastics used for this specific application [12,13].

A major concern for traditional thermoplastics used in packaging applications refers to the non-renewability of the raw materials employed for their production, which are commonly derived from fossil fuels through refining processes [14,15]. Additionally, the majority of the typical fossil fuel-based polymers are non-biodegradable, thus causing environmental issues related to their landfill confinement [16].

Due to the rising attention towards the environmental safety and the consequent increasing demand of sustainable products, in recent years both academic and industrial research focused on the development of new bio-sourced polymers suitable for packaging applications [17,18,19]. In this context, polylactic acid (PLA) is the most exploited bio-based and biodegradable polymer, due to its high transparency, high water resistance and good processability, notwithstanding that its mechanical and thermal properties result often insufficient for certain packaging applications [20,21]. Besides, biopolymers belonging to the MaterBi^®^ family are attracting a steadily increasing interest in the last years as bio-sourced thermoplastics suitable for replacing traditional fossil fuel-based counterparts, especially in the formulation of films for the packaging industry [22,23,24]. MaterBi^®^ products are heterogeneous systems with proprietary composition, based on modified starch and synthetic bio-based polymers [25], and exhibit good performances in terms of mechanical properties, thermal stability, and biodegradability [26,27].

Film blowing is the most common method exploited for formulating polymer-based films for packaging applications [28]; however, this kind of processing is often challenging for biopolymers, due to their typical low melt strength and reduced elongation as compared to standard polyolefins [29,30], resulting in unstable and wrinkled bubbles that tend to collapse during the processing operations [31]. Different strategies aiming at overcoming this issue have been proposed, including the use of viscosity enhancers to improve the material melt strength [32], or the introduction of different kinds of fillers, able to concurrently improve the processability and the barrier properties of the produced films [33,34]. In this context, Thellen et al. [35] demonstrated the effectiveness of incorporating organo-modified clay particles into PLA for obtaining a blown film with enhanced mechanical and barrier properties, as compared to the unfilled polymer. Interestingly, Herrera et al. [36] investigated bionanocomposite films obtained through film blowing and based on PLA and chitin nanocrystals, documenting a beneficial effect of the embedded nanofillers on the mechanical and optical properties of the polymer matrix; furthermore, the bionanocomposite films exhibited lower electrostatic interactions between the film surfaces, resulting in easier opening of the plastic bags.

In this work, bionanocomposites based on different MaterBi^®^ polymers and two kinds of modified clays were first compounded in a twin-screw extruder, and then subjected to a film blowing process. A systematic rheological characterization of the materials, both in shear and in non-isothermal elongational flow was performed, aiming at obtaining a detailed assessment of the bionanocomposite processing behavior; furthermore, the mechanical behavior of the resulting blown films was assessed. This way, it was possible to identify the most promising systems suitable for upscaled film blowing operations.

## 2. Materials and Methods

### 2.1. Materials

In this work, two different types of Mater Bi^®^ (Novamont Spa, Novara, Italy) biopolymers based on blends of aliphatic and aromatic biodegradable co-polyesters with proprietary composition were used as polymeric matrices, namely:

- HF03V0 (hereafter coded as MB1): density = 1.28 g/cm^3^, Melt Flow Index - MFI (160 °C, 2.16 kg) = 2.5 g/10 min;
- EF51L (hereafter coded as MB2): density = 1.22 g/cm^3^, MFI (190 °C, 2.16 kg) = 4.5 g/10 min;

Furthermore, a low-density polyethylene (LDPE) FT 3200 from Borealis (Vienna, Austria), suitable for film extrusion (density = 920 kg/m^3^, MFI (190 °C, 2.16 kg) = 0.25 g/10 min) was used as reference material.

Two kinds of modified clays were employed for the formulation of bionanocomposites, namely Cloisite 20A (hereafter coded as CL20A, from Southern Clay Product, Gonzales, TX, USA, is a ditallow dimethyl ammonium-modified montmorillonite with particle size < 10 μm) and BYK 02 BLOCK 1200 (hereafter coded as BYK1200, BYK-Chemie GmbH, Wesel, Germany, a modified aluminum-magnesium clay designed for the introduction on biopolymers-based films, showing particle size < 50 μm).

### 2.2. Nanocomposite and Blown Film Preparation

The preparation of all investigated nanocomposites was carried out in a co-rotating twin-screw extruder (OMC, Saronno, Italy); the processing conditions, i.e., temperature profile and screw rotation speed were suited for each polymer matrix and are listed in Table 1. Prior to the extrusion, all used polymers and nanofillers were vacuum-dried at 60 °C for 5 h and at 120 °C for 12 h, respectively [37].

Each nanofiller was added at 5 wt % in the polymer matrix.

Specimens for rheological and mechanical characterizations were produced through a compression molding step, using a Carver laboratory press working at a pressure of 100 bar; the temperatures selected for compression molding correspond to each single temperature achieved in the die during the extrusion process, namely 150 °C for nanocomposites based on MB1, 145 °C for nanocomposites based on MB2 and 180 °C for nanocomposites based on LDPE.

Then, the extruded nanocomposites were subjected to a film blowing process, performed in a single screw (D = 19 mm, L/D = 25) extruder (Brabender, Duisburg, Germany) equipped with a film blowing head and a take-off unit. The selected processing conditions are listed as follows: temperature profile 120-130-140-150-160-170; screw rotation speed 80 rpm; draw ratio (DR) 3; blow up ratio (BUR) 2.

For comparison purposes, the unfilled polymers were subjected to the same processing.

### 2.3. Characterizations

The dynamic rheological behavior of unfilled matrices and all investigated nanocomposites was assessed through frequency sweep tests using an ARES G2 rheometer (TA instruments, New Castle, DE, USA) in parallel plate geometry (gap diameter = 25 mm) from 10^−1^ to 10^2^ rad/s. The strain amplitude was fixed at 5%, which is within the linear viscoelastic region (as established from preliminary strain sweep tests).

Rheological characterization in shear flow was performed using a capillary rheometer Rheologic 1000 (Ceast, Torino, Italy) with a capillary having D = 1 mm and L/D = 40.

The same capillary rheometer, equipped with a tensile drawing unit, was exploited for assessing the rheological behavior of all the investigated materials in non-isothermal elongational flow. Melt strength (MS) and breaking stretching ratio (BSR) of the samples were measured; more specifically, MS refers to the force in the molten filament at breaking, while BSR is the ratio between the drawing speed at breaking and the extrusion rate.

The temperatures selected for the rheological characterization of the different materials correspond to each single temperature achieved in the die during the extrusion process, namely 150 °C for nanocomposites based on MB1, 145 °C for nanocomposites based on MB2 and 180 °C for nanocomposites based on LDPE.

Mechanical analyses were performed on both compression molded samples and blown films (both in machine and transverse direction) using a Zwick/Roell dynamometer (Zwick/Roell Z005, ZwickRoell S.r.l., Genova, Italy) with a crosshead speed of 50 mm/min. At least five specimens for each investigated system were tested and the results averaged.

Morphological characterization was performed through SEM analysis, using a Philips ESEM XL 30 (Philips, Milano, Italy), on fracture surfaces of gold-sputtered samples.

## 3. Results and Discussion

### 3.1. Morphology

**Figure 1:**
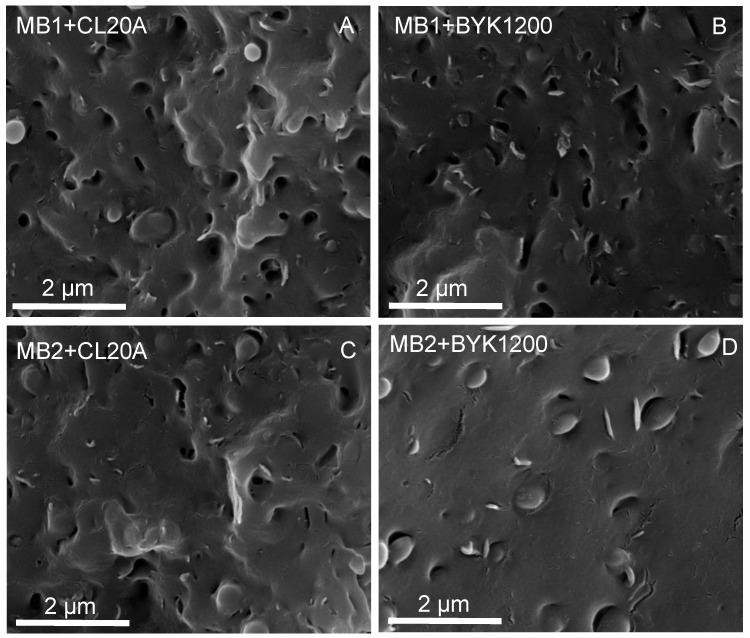
SEM micrographs of (**A**,**B**) MB1- and (**B**,**C**) MB2-based bionanocomposites.

The morphology of the extruded bionanocomposites was evaluated through SEM observations, aiming at assessing the extent of the nanofiller dispersion within the selected biopolymeric matrices. The typical micrographs are presented in Figure 1: they clearly indicate the intrinsic multi-phase structure of both biopolymers. More specifically, either MB1 or MB2 are based on blends of aromatic and aliphatic biopolyesters, showing the typical droplet-matrix morphology of immiscible or partly miscible polymer blends. Irrespective of the matrix, all the investigated bionanocomposites exhibit a homogeneous dispersion of both types of modified nanoclays; in particular, the embedded nanofillers seem to be dispersed at sub-micrometric scale as tactoids, suggesting the formation of intercalated structures. Furthermore, the absence of pull-out phenomena involving the embedded nanofillers indicate the achievement of a high extent of biopolymer–filler interaction in the interfacial region, resulting in a good interfacial adhesion between the two phases.

### 3.2. Rheological Behavior

**Figure 2:**
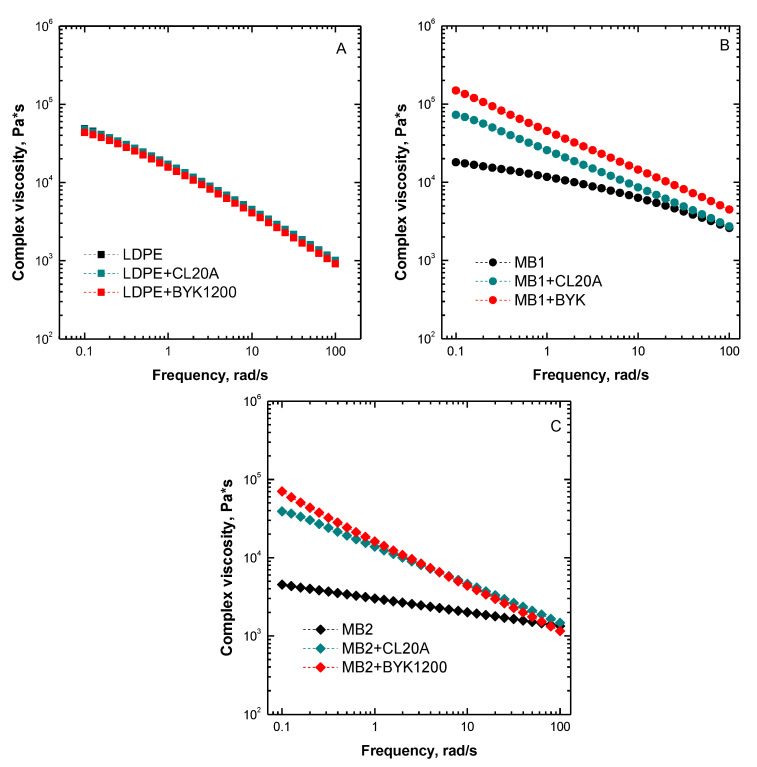
Complex viscosity curves as a function of frequency for (**A**) LDPE-, (**B**) MB1-, and (**C**) MB2-based nanocomposites. The complex viscosity trends for the unfilled matrices are also reported.

Figure 2A–C depicts the complex viscosity trends as a function of frequency for all the investigated nanocomposites and unfilled matrices. As far as LDPE-based nanocomposites are concerned, the complex viscosity curves reported in Figure 2A clearly indicate a marginal effect of the introduced nanofillers on the rheological behavior of the polyolefin. In fact, the curves of the nanocomposites containing CL20A and BYK1200 nanofillers are almost overlapped with that of the unfilled polymer, indicating the establishment of weak interactions between the polymer chains and the embedded nanoclays in the interfacial region.

Conversely, the bionanocomposites containing either CL20A or BYK1200 nanofillers exhibit a remarkably different rheological behavior as compared to the corresponding unfilled matrices. In particular, irrespective of the biopolymeric matrix, the incorporation of both types of nanoclays induces higher viscosity values as compared to the unfilled biopolymer; this behavior is more pronounced in the lowest investigated frequency range. Furthermore, the appearance of a markedly non-Newtonian behavior, involving the occurrence of an apparent yield stress in the low frequency region and the amplification of the shear thinning behavior at higher frequencies can be observed. The rheological response of all investigated bionanocomposites suggests that the relaxation processes of the biopolymer chains are strongly affected by the presence of the embedded nanofillers [38]. More specifically, the well dispersed nanoclay tactoids are able to interfere with the dynamics of the macromolecules, thus hindering their complete relaxation.

**Figure 3:**
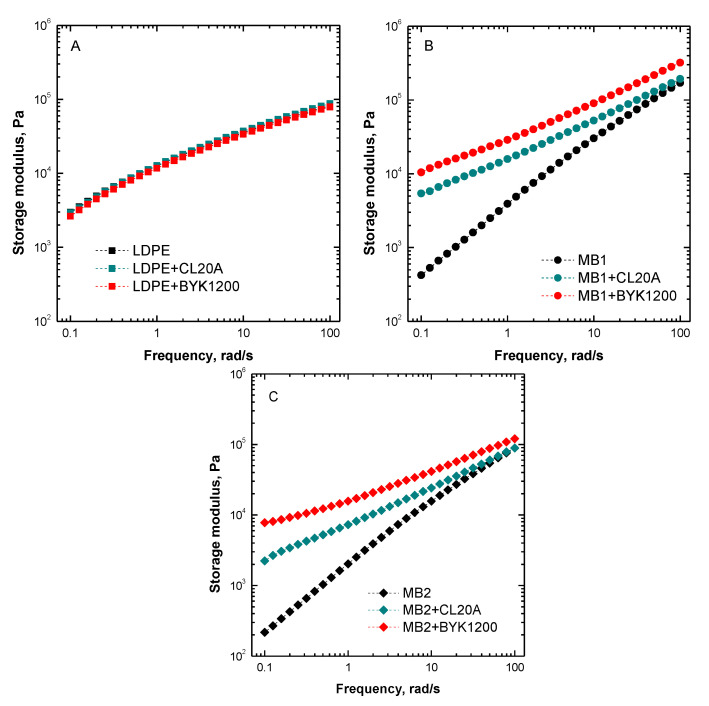
Storage modulus as a function of frequency for (**A**) LDPE-, (**B**) MB1-, and (**C**) MB2-based nanocomposites. The moduli trends for the unfilled matrices are also reported.

Similar conclusions can be drawn from the evaluation of the trend of the storage modulus as a function of frequency plotted in Figure 3A–C. In fact, similarly to what observed for the complex viscosity trends, the rheological behavior of LDPE nanocomposites is almost coincident with that of their unfilled counterpart, highlighting the poor level of interactions achieved in these systems. At variance, the storage modulus trends exhibited by MB1- and MB2-based bionanocomposites confirm the significant modification of the biopolymer rheological response resulting from the introduction of both types of nanoclays. More specifically, as compared to the respective matrix, bionanocomposites show significantly higher modulus values in the low frequency region, along with a dramatic variation of the frequency dependence of the moduli curves. In fact, the bionanocomposites exhibit a well pronounced solid-like behavior, recognizable in the decrease of the slope of the moduli trends in the terminal region; this behavior can be attributed to the formation of an interconnected polymer-nanofiller and nanofiller-nanofiller network, strongly hampering the macromolecule dynamics [39].

Usually, as well documented for polymer-based nanocomposites containing layered nanofillers, the observed modifications of the rheological behavior of the MB1 and MB2 biopolymers resulting from the introduction of both types of nanoclays are associated with the formation of intercalated structures [40,41]. In fact, the introduction of the polymer chains within the inter-layer galleries and their consequent immobilization, induces a retardation on their dynamic response and a consequent incomplete relaxation [42]. Additionally, the higher complex viscosity and storage modulus values displayed by the bionanocomposites can be ascribed to the increased biopolymer/nanofiller interface achieved as a result of intercalation phenomena [43].

**Figure 4:**
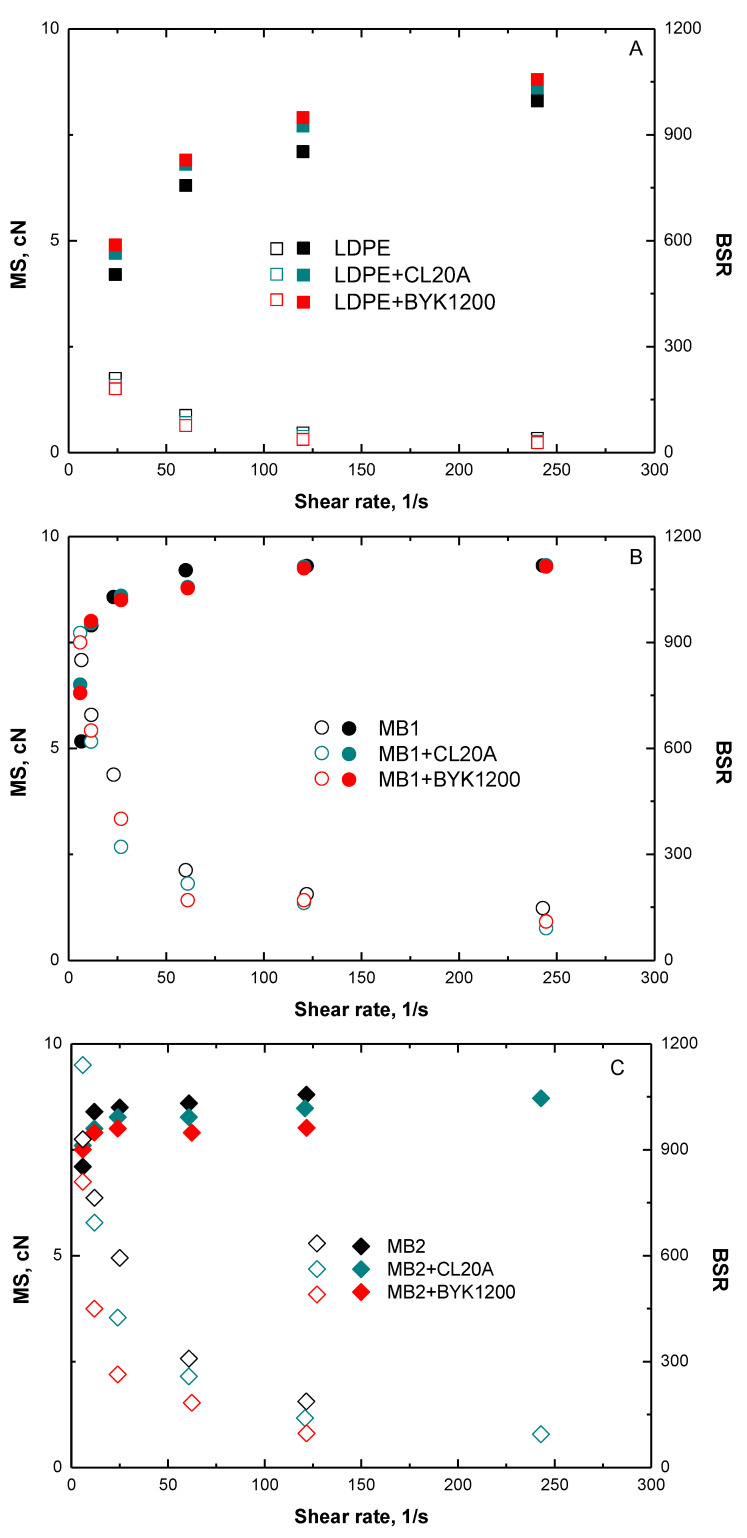
MS (filled symbols) and BSR (blank symbols) values as a function of shear rate for (**A**) LDPE-, (**B**) MB1-, and (**C**) MB2-based nanocomposites. The values for the unfilled matrices are also reported.

Since the main purpose of the present study is the formulation of biopolymer-based blown films, the assessment of the rheological behavior of the investigated systems under non-isothermal elongational flow is fundamental to gain important information about the material filmability. Therefore, all the investigated nanocomposites and unfilled polymers have been characterized in order to evaluate the values of melt strength and breaking stretching ratio and the obtained results as a function of the shear rate are plotted in Figure 4. First, worth noting that both unfilled MB1 and MB2 show similar MS and higher BSR values as compared to LDPE, indicating the suitability of both selected biopolymers for film blowing operations. From a general point of view, all investigated nanocomposites exhibit a behavior quite similar to that of their unfilled counterparts, thereby indicating that the introduction of the nanofillers does not negatively affect the filmability of the materials. In particular, LDPE- and MB1- based nanocomposites show slightly enhanced MS values with respect to the respective matrix and unchanged deformability, confirming the achievement of a uniform dispersion of nanoclays within the host matrices and a good interfacial adhesion between the two phases. Interestingly, nanocomposites based on MB2 exhibit a decreased MS compared to the unfilled biopolymers; this finding can be related to the occurrence of a solid-like rupture during the application of the elongational flow, involving the premature break of the material, which is not able to reach the expected stress level due to its limited deformability [44].

### 3.3. Mechanical Properties

**Figure 5:**
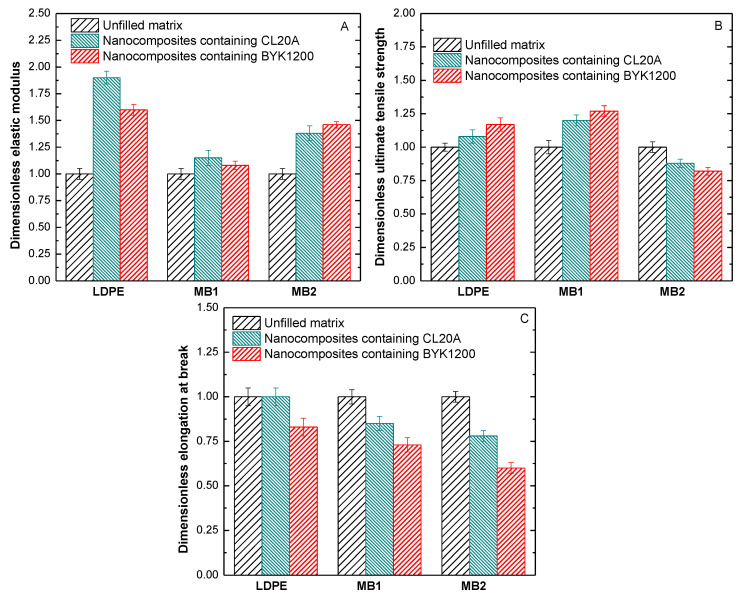
Dimensionless (**A**) elastic modulus, (**B**) ultimate tensile strength, and (**C**) elongation at break for all investigated materials.

Tensile tests were exploited to evaluate the mechanical properties of the formulated nanocomposites; the main results in terms of elastic modulus (E), ultimate tensile strength (UTS), and elongation at break (EB) are listed in Table 2. Furthermore, to better elucidate the effect of the introduced nanoclays on the tensile properties, the dimensionless values of the main tensile properties were calculated by dividing the value of the property by that of the corresponding unfilled matrix; the resulting values are reported in Figure 5.

The general behavior of all investigated bionanocomposites involves the achievement of enhanced values of the elastic modulus (Table 2 and Figure 5A) with respect to the unfilled counterparts, due to the uniform dispersion of the nanoclays and to the formation of intercalated structures, further increasing the material stiffness. As far as the values of ultimate tensile strength are concerned (Table 2 and Figure 5B), nanocomposites based on LDPE and MB1 exhibit improved values as compared to the unfilled matrices; differently, the introduction of both types of nanoclays causes a decrease of the UTS values for MB2-based systems. This behavior can be associated with the low ductility of the bionanocomposites (Table 2 and Figure 5C) as compared to the unfilled matrix, inducing a premature failure of the samples during the test [45].

### 3.4. Processing Behavior

Aiming at evaluating the suitability of the formulated bionanocomposites for the production of blown films, the processing behavior of the materials was assessed and compared to that of LDPE, which represents the standard material usually employed at industrial level for the production of blown films.

**Figure 6:**
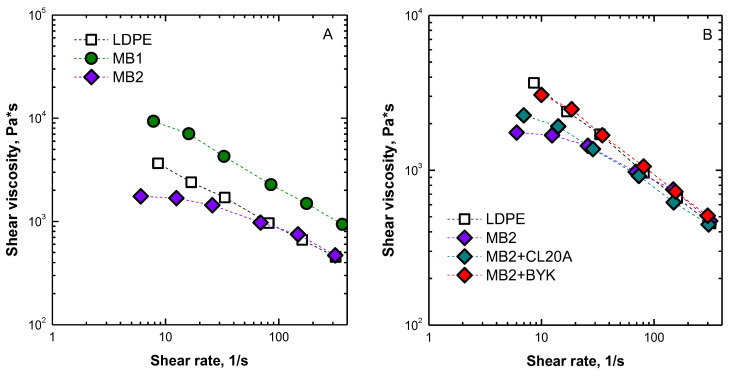
Shear viscosity as a function of shear rate for (**A**) unfilled polymers and (**B**) unfilled LDPE and MB2 and MB2-based bionanocomposites.

First, the rheological characterization of the unfilled biopolymers was performed in the shear rate range typically involved in an industrial film blowing operation. Looking at the flow curves reported in Figure 6A, it is evident that MB2 exhibits a rheological behavior very similar to that of LDPE, indicating that the processability of this biopolymer matches that of the standard material. Differently, MB1 shows higher viscosity values as compared to LDPE in the shear rate range of interest. Furthermore, the rheological characterization performed on MB2-based nanocomposites suggests that the introduction of either CL20A or BYK1200 nanofillers does not significantly modify the rheological behavior of the unfilled matrix. In fact, as observable from the flow curves reported in Figure 6B, the shear viscosity values of the MB2-based bionanocomposites are almost unchanged as compared to those of the unfilled biopolymer, highlighting that the processability of MB2 biopolymers is not negatively affected by the presence of embedded nanoclays.

### 3.5. Characterization of Blown Films

Based on the previous considerations about the material processability, unfilled MB2 and MB2-based nanocomposites were selected for the production of blown films, through a further processing step in a single screw extruder equipped with a film blowing head and a take-off unit. Samples derived from the as-produced films were then characterized through tensile tests, to verify if the selected materials fulfill the requirements in terms of mechanical performances for film blowing applications. Table 3 collects the results obtained for unfilled MB2 and MB2-based composites, compared to those of a standard LDPE film subjected to the same processing. The tensile properties of LDPE and MB2 films documented the achievement of higher elastic modulus and ultimate tensile strength values as compared to the isotropic as-extruded materials (whose tensile properties are shown in Figure 5). This finding can be associated to the effect of the elongational flow experienced by the materials during the processing, which induces a preferential orientation of the polymer macromolecules along the flow direction [23]. In particular, for both unfilled polymers, higher values of elastic modulus were obtained in the machine direction than in the transverse one, suggesting a higher degree of orientation for LDPE- and MB2-based films in this direction, as also confirmed by their lower ductility evaluated in the same direction.

A quite similar behavior was observed for MB2-based bionanocomposites; in fact, the application of the elongational flow during the film blowing process induces an enhancement of the elastic modulus and ultimate tensile strength of the materials, as compared to the as-extruded samples. In this case, the improvement of the tensile performances can be explained considering, apart the partial orientation of the biopolymeric chains promoted by the elongational flow, also the possible orientation of the embedded nanoclays along the flow direction. Differently to what observed for the unfilled polymers, in the case of the bionanocomposites the variation of the tensile properties with respect to the as-extruded samples is similar in machine and transverse directions, indicating that the drawing in the machine direction and the blowing in the transverse direction occur simultaneously during processing [33].

Interestingly, the bionanocomposite film containing BYK1200 nanofillers shows unchanged or slightly higher ductility with respect to the as-extruded isotropic sample; this unusual behavior has been already observed for nanocomposites containing layered nanofillers, and associated with a facilitated deformation mechanism of the polymer macromolecules in presence of nanoclay layers oriented along the same direction [26,46].

## 4. Conclusions

In this work, bionanocomposites based on two different biopolymers and two types of modified nanoclays were produced through melt extrusion, and their morphology, rheological, and mechanical behavior were evaluated and compared to those of similar materials based on a LDPE matrix. SEM observations proved the obtainment of a uniform nanofiller dispersion within the selected biopolymeric matrices; furthermore, rheological characterization indicated that the incorporation of both types of nanoclays remarkably affects the low-frequency rheological response of the materials, while does not promote significant modification of the rheological behavior of the correspondent matrices under non-isothermal elongational flow. The assessment of the processing behavior of all investigated materials allowed selecting unfilled MB2 and MB2-based bionanocomposites as the most suitable systems to be processed through a film blowing unit; the obtained blown films exhibited mechanical performances suitable for their possible exploitation as materials for the packaging industry, in alternative to traditional fossil fuel-based thermoplastics.

## Figures and Tables

**Table 1:** Processing conditions in the twin screw extruder for the formulation of all the nanocomposites.

Polymer Matrix	Temperature Profile (°C)	Screw Rotation Speed (rpm)
MB1	90-11 -130-140-140-150-150	205
MB2	90-110-130-140-140-145 -145	220
LDPE	110-120-140-160-170-180-180	220

**Table 2:** Main mechanical properties of all investigated materials.

	Elastic Modulus (E) (Pa)	Ultimate Tensile Stress (UTS) (MPa)	Elongation at Break (EB) (%)
LDPE	80 ± 8.1	12.0 ± 3.2	613 ± 48
LDPE + CL20A	152 ± 19.1	12.9 ± 0.5	615 ± 47
LDPE + BIK1200	128 ± 12	14.1 ± 3	512 ± 38
MB1	111 ± 6.5	13.4 ± 3.4	475 ± 52
MB1 + CL20A	127 ± 8.4	16.1 ± 0.7	406 ± 50
MB1 + BYK	120 ± 9.0	16.9 ± 3.3	350 ± 20
MB2	105 ± 14.2	17 ± 1.6	620 ± 10
MB2 + CL20A	145 ± 17.0	15.0 ± 1.5	487 ± 19
MB2 + BYK	153 ± 19.4	14.2 ± 0.8	378 ± 15

**Table 3:** Main tensile properties in machine direction (MD) and transverse direction (TD) of LDPE, unfilled MB2 and MB2-based bionanocomposite blown films.

	MD			TD		
	E (Pa)	UTS (MPa)	EB (%)	E (Pa)	UTS (MPa)	EB (%)
LDPE	170 ± 10.1	26 ± 1.8	350 ± 20	160 ± 12.1	27 ± 1.9	540 ± 14
MB2	320 ± 7.2	31.5 ± 2.0	316 ± 20	275 ± 9.9	27.5 ± 1.8	467 ± 22
MB2 + CL20A	398 ± 7.0	34.5 ± 1.1	352 ± 18	390 ± 8.5	32.2 ± 1.7	402 ± 15
MB2 + BYK	452 ± 9.3	39.5 ± 0.6	420 ± 15	462 ± 10.1	31.5 ± 1.3	435 ± 25

## Data Availability

The data presented in this study are available on request from the corresponding author.
